# DNA repair inhibitors sensitize cells differently to high and low LET radiation

**DOI:** 10.1038/s41598-021-02719-9

**Published:** 2021-12-01

**Authors:** Kristina Bannik, Balázs Madas, Sabrina Jarke, Andreas Sutter, Gerhard Siemeister, Christoph Schatz, Dominik Mumberg, Sabine Zitzmann-Kolbe

**Affiliations:** 1 Pharmaceuticals Division, Bayer AG, Berlin, Germany; 2 Centre for Energy Research, Budapest, Hungary; 3 Berlin, Germany; 4 Present Address: Nuvisan-ICB GmbH, Berlin, Germany

**Keywords:** Cell biology, Cancer, Cancer therapy, Radiotherapy

## Abstract

The aim of this study was to investigate effects of high LET α-radiation in combination with inhibitors of DDR (DNA-PK and ATM) and to compare the effect with the radiosensitizing effect of low LET X-ray radiation. The various cell lines were irradiated with α-radiation and with X-ray. Clonogenic survival, the formation of micronuclei and cell cycle distribution were studied after combining of radiation with DDR inhibitors. The inhibitors sensitized different cancer cell lines to radiation. DNA-PKi affected survival rates in combination with α-radiation in selected cell lines. The sensitization enhancement ratios were in the range of 1.6–1.85 in cancer cells. ATMi sensitized H460 cells and significantly increased the micronucleus frequency for both radiation qualities. ATMi in combination with α-radiation reduced survival of HEK293. A significantly elicited cell cycle arrest in G_2_/M phase after co-treatment of ATMi with α-radiation and X-ray. The most prominent treatment effect was observed in the HEK293 by combining α-radiation and inhibitions. ATMi preferentially sensitized cancer cells and normal HEK293 cells to α-radiation. DNA-PKi and ATMi can sensitize cancer cells to X-ray, but the effectiveness was dependent on cancer cells itself. α-radiation reduced proliferation in primary fibroblast without G_2_/M arrest.

## Introduction

Radiation still remains the most widely utilized treatment modality in the clinical management of cancer^1^. Tumors respond to radiation-induced DNA damage by activating a complex network of DNA damage signaling and repair pathways that determine each cell’s fate which is survival or death, and determines genome stability^2–4^. The agents that inhibit these key DNA damage response proteins are currently being combined with conventional radiotherapy and genotoxic chemotherapy in clinical trials in the hope of complementing each other’s strengths and effects. However, much less is known about these combination possibilities with alpha radiation. Radium-223 dichloride (Ra-223) is the first approved targeted alpha therapy (TAT). Ra-223 monotherapy in metastatic prostate cancer reported an overall survival benefit of 3.6 months compared to placebo (phase III ALpharadin in SYMPtomatic prostate CAncer patients (ALSYMPCA) study^5^. Interestingly, in this patient group with metastatic prostate cancer, the prevalence of germline alterations in DNA repair genes (*BRCA1*, *BRCA2*, and *ATM*) may be as high as 11.8%^6^. Analysis of somatic and Germline aberrations in metastatic, castration-resistant prostate cancer (mCRPC) showed (1) the most frequently aberrant gene is *TP53* (53.3%), (2) alterations in *BRCA2* identified 19/150 (12.7%) of cases with loss of *BRCA2*, of which 90% exhibited biallelic loss, (3) other DNA repair/recombination genes (*BRCA1, CDK12, FANCA, RAD51B,* and *RAD51C*) and identified alterations in at least 34/150 (22.7%) of cases^7,8^.

For this study, Ra-223 was used as a tool compound to investigate the in vitro effects of alpha particle radiation. Ra-223 is an α-emitter which induces complex DNA double strand breaks (DSBs) in cells. DSBs can be repaired at least by three pathways—homologous recombination (HR), non-homologous end joining (NHEJ), and alternative end joining. Although repair of complex DNA lesions induced by high LET radiation is still not well understood, it has been proposed that complex DSBs generated by high LET irradiation are repaired by HR and not NHEJ in mammalian cells^9^. However, recent reports have indicated that NHEJ may play a prominent role in repair of carbon ion-induced damage^10,11^. Three members of the family of phosphatidyl inositol 3-kinase-like kinases (PIKKs) are central to the DSB response: Ataxia-telangiectasia mutated (ATM), ataxia telangiectasia and Rad3-related (ATR) and DNA-dependent protein kinase (DNA-PK). DNA-PK plays a direct role in classical NHEJ^12^. ATM is important for the detection, signaling and repair of DSBs, which are the most cytotoxic DNA lesions induced by ionizing radiation (IR) and certain chemotherapies^13,14^ whereas ATR functions mainly in the protection of single stranded DNA upon replication stress^15^. Inhibitors of these three PIKKs are now in phase I/II clinical trials as single agent or in combination treatments.

Cheng et al.^16^ investigated sensitizing effects of inhibitors of ATM and DNA-PK kinase activity towards alpha and gamma radiation in primary lymphocytes, however little is known about the combination effects on human tumor cells. Here, we compare the effects of α-radiation from Ra-223 or X-ray radiation upon combination with the DNA-PK inhibitor M3814^17^ and the ATM inhibitor AZD-1390^18^ in several cell lines (tumor cell lines, normal cell lines and one immortalized cell line). Cellular parameters such as cell cycle distribution, clonogenic cell survival and micronucleus formation were investigated to determine the cellular effects of the combination of DDR inhibitors and radiation.

## Materials and methods

### Cell culture and reagents

Cell lines were obtained from the American Type Culture Collection and from the German Collection of Microorganisms and Cell Cultures. Cells were propagated in RPMI-1640 medium (H460, 22Rv1, HCT116, A549, H1299, MRC-5 and HEK293), in MEM Eagle’s (CCD 1096Sk and IMR-90) supplemented with 10% fetal bovine serum (ThermoFischer, MA) and 1% Antibiotic Antimycotic solution (Sigma). All cultures were incubated at humidified 37 °C in 5% CO_2_. M3814 and AZD-1390 were synthesized at Bayer AG.

### Dosimetry/radiation

Cells were irradiated in a Transwell^®^ system (TW) consisting of a culture plate and an insert with membrane. The cells were seeded onto the membrane of the various Transwell^®^ systems (3450, 3460 or 3470, Corning). Growth of the cells on the insert membrane was tested and compared with growth on corresponding well plates. Radiation of the cells in the TWs was performed by X-rays delivered at 1.5 Gy/min with an Al 0.5 mm filter using a CellRad equipment (Faxitron, USA). The exposure to α-particles was performed by coating the bottom of a TW with Ra-223. To achieve an even coating, Ra-223 (Xofigo^®^, Bayer AG, Germany) in its ionic form was mixed with a 70%—ethanol and was dried overnight. The cells were seeded on a 10-µm-think, transparent polyester membrane 24 h prior to radiation and irradiated with α-particles from the bottom of the wells through the membrane. For the Monte–Carlo simulations, we first determined the number of alpha-decays with the initial activities and the duration of exposure. The same number of alpha-particles have been simulated for the four radionuclides with alpha decays. The location of radioactive decays has been selected randomly using a uniform distribution over the area of the bottom well. The direction of movement has been selected randomly in the 4 PI solid angle. If the alpha-particle crosses the top of the polyester membrane, then we calculate the location where it crossed the bottom of the membrane, and the distance traveled within the air. The energy losses in the air, in the polyester membrane and in the cells are calculated using the SRIM code^19^ considering the distance traveled in the different media. If the energy of the alpha-particle is larger than zero after crossing the polyester membrane, then hits and doses are calculated for ellipsoid shaped cell nuclei which are located in concentric rounds in the upper well. This configuration of cell nuclei represents the hit and dose distribution precisely, because the average number of hits and average dose do not depend on the distance from the centre of the upper well. Procedure also described in Bannik et al.^20^.

### Cell characteristics

The cell morphology was determined on live cells using an Operetta CLS™ high-content analysis system, non-confocal. Cell thickness measurement was performed by adding Hoechst 33342. The cell size data were used in the Monte Carlo simulations. Procedure also described in Bannik et al.^20^.

### Cell cycle distribution

The cells were plated into Transwell inserts (3450, Corning) with a density of 0.2 × 10^6^ cells/well. After irradiation, the cells were collected by trypsin into tubes, washed with PBS and fixed with 70% Ethanol overnight at − 20 °C. For the cell cycle analyses, the cells were washed with PBS and treated with RNaseA (Sigma, R-4875, 5% in PBS) and stained with propidium iodide (PI) (Sigma, P-4170, 100 µg/ml in PBS) for 2 h at 4 °C. The samples were measured with FacsCalibur 3CS (Becton Dickinson, USA). The results were analyzed by using software CellQuest. Procedure also described in Bannik et al.^20^.

### Cell survival

The cells were plated into Transwell inserts (3460, Corning) with a density of 0.1 × 10^6^ cells/well. Next day the cells were irradiated with α-particles from Ra-223 for different periods of time or with X-rays. The cells were treated with DDR inhibitors 2 h prior to radiation. After irradiation, the inserts were incubated for an additional 24 h. The cells were then detached and seeded into new 6-well plates in triplets and incubated for 9–30 days for colony formation, depending on the cell growth rate. A colony was defined as consisting of at least 50 cells. The colonies were fixed with 11% Glutardialdehyd (Merck) for 20 min and stained with ten times diluted crystal violet solution (Sigma). The colonies were counted manually and plating efficiency and survival were calculated.

### In vitro micronucleus test

The cells were seeded into Transwell inserts (3470, Corning) at a density of 3 × 10^3^ cells/well. Next day, the cells were treated with DDR inhibitors for 2 h prior to α-radiation or X-rays. The cells were irradiated with the activities of 1.3 kBq/cm^2^ for 2, 4, 8 h, resulting in absorbed doses of 4, 8 and 16 Gy. After irradiation the cells were incubated for 24 h (22Rv1 for 48 h) and then placed on ice for 20 min. Subsequently, the micronucleus test was performed according to the In Vitro MicroFlow^®^ Kit manual (Litron Laboratories, Rochester, NY). In brief, the cells were stained with Nucleic Acid Dye A (containing ethidium monoazide, EMA) and were exposed to visible light for 30 min on ice. The cells were washed up with a buffer solution and incubated in lysis solution 1 with Nucleic Acid Dye B (containing SYTOX-Green) for one hour in the dark at 37 °C. After incubation, the lysis solution 2 was added into wells and the samples were analyzed in triplets within 24 h by FACS (MACS Quant 10 flow cytometer, Miltenyi GmbH, Bergisch Gladbach, Germany).

### Statistical analysis

All experiments were performed at least two times. Numerical data were analyzed using two-way ANOVA test with Tukey’s multiple comparisons for survival and Dunnett's multiple comparisons test for the micronucleus test in GraphPad Prism 8. Significance thresholds are indicated in legends.

## Results

In our work we compared the radiobiological effects of combining DNA damage response (DDR) inhibitors (DNA-PK and ATM) to alpha particle high LET radiation and X-ray low LET radiation in different cell lines. Since alpha particles have only a very limited range (~ 100 µm in water in case of 10 MeV), they can easily be shielded by water or plastic. Therefore, we applied the Transwell system to deliver the alpha particles to the cells and to measure and calculate the absorbed dose in the cell nuclei.

### Absorbed dose calculation

In order to calculate the absorbed dose delivered by the α-radiation source to the cells in our experimental set up, first the size of cells and nuclei were measured for H460, A549, H1299 HCT116 cancer cell lines, the immortalized HEK293 cell line and CCD 1096Sk, IMR-90, and MRC-5 normal cells (Table 1) by microscopy. Then Monte-Carlo simulations were applied to calculate the average absorbed dose and its standard deviation for individual cell nuclei. Using the conditions of 2–8 h exposure to a radioactivity of 1.32 kBq/cm^2^ in our set up, the absorbed doses of in the observed cell lines were very similar.

**Table 1:** Cell size and absorbed doses.

	Cell length (μm)	Cell width (μm)	Cell height (μm)	Nucleus length (μm)	Nucleus width (μm)	Nucleus height (μm)
H460	20	16	9	12.5	8.5	7
A549	19.4	10	8.4	18	9.3	5.8
H1299	20.2	18	15.6	13	9	8.8
HCT116	18	12	13.5	11.5	8.3	9.6
CCD-1096Sk	52.3	19.2	9.3	15.9	11.9	5.2
IMR-90	51.4	19	8	18.8	10.4	6.8
MRC-5	33.9	14	12.8	14.4	10.5	8.1
HEK293	16.2	8	17.1	15.9	11.9	8.1

Cell line	2 h	4 h	6 h	8 h		
H460	4.1 ± 1.3 Gy	8.2 ± 1.7 Gy	12.2 ± 2.1 Gy	16.2 ± 2.4 Gy		
A549	4.1 ± 1.1 Gy	8.4 ± 1.5 Gy	12.5 ± 1.8 Gy	16.6 ± 2.1 Gy		
H1299	4.0 ± 1.1 Gy	7.9 ± 1.5 Gy	11.8 ± 1.9 Gy	15.8 ± 2.1 Gy		
HCT116	3.9 ± 1.3 Gy	7.8 ± 1.9 Gy	11.8 ± 2.2 Gy	15.5 ± 2.7 Gy		
CCD-1096Sk	4.3 ± 1.3 Gy	8.4 ± 1.5 Gy	12.6 ± 1.9 Gy	16.9 ± 2.0 Gy		
IMR-90	4.1 ± 1.0 Gy	8.2 ± 1.3 Gy	12.3 ± 1.6 Gy	16.3 ± 1.8 Gy		
MRC-5	3.9 ± 1.0 Gy	8.0 ± 1.3 Gy	12.0 ± 1.7 Gy	15.9 ± 2.1 Gy		
HEK293	4.0 ± 0.9 Gy	7.9 ± 1.3 Gy	12.1 ± 1.5 Gy	16.0 ± 1.8 Gy		

Therefore, the average dose delivered to all cell lines was used for all cell lines in this paper.

### Effect of DDR inhibition on cell survival and the sensitization enhancement ratio after alpha radiation and X-ray

**Figure 1:**
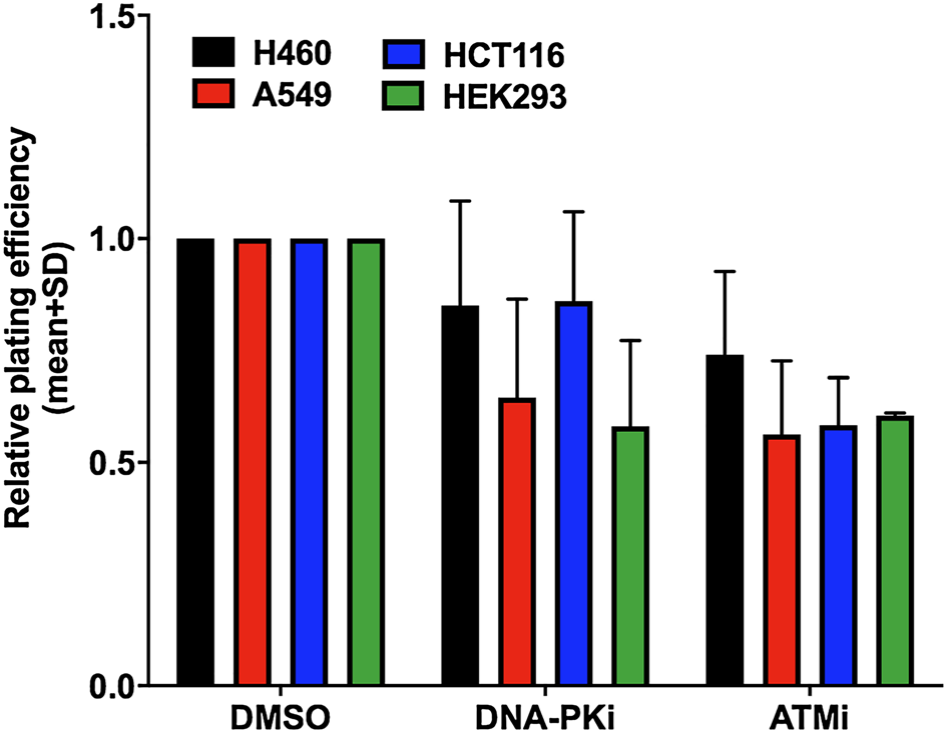
Reduction of plating efficiency after DDR inhibitor treatment in various cancer cells.

The cancer cells A549, H460 lung, HCT116 colon cancer cells and immortalized kidney HEK293 cells were treated with DNA-PKi (M3814) or ATMi (AZD1390), then incubated for 24 h and re-seeded for colony formation. It has to be noted that the fibroblast cells (CCD 1096Sk, IMR-90 and MRC-5) were not able to form colonies. In order to investigate DNA damage response inhibition as single treatment, plating efficiency was evaluated and normalized to DMSO control. Both DDR inhibitors reduced the plating efficiency (PE) alone in all cell lines. For DNA-PKi (M3814) or ATMi (AZD1390) a concentration of 200 nM was selected (see “Suppl.”), as we wanted to work in the frame of radiation-induced effects and tried to use non-toxic concentrations of the inhibitors. The plating efficiency at the selected DDRi concentration is shown in Fig. 1.

**Figure 2:**
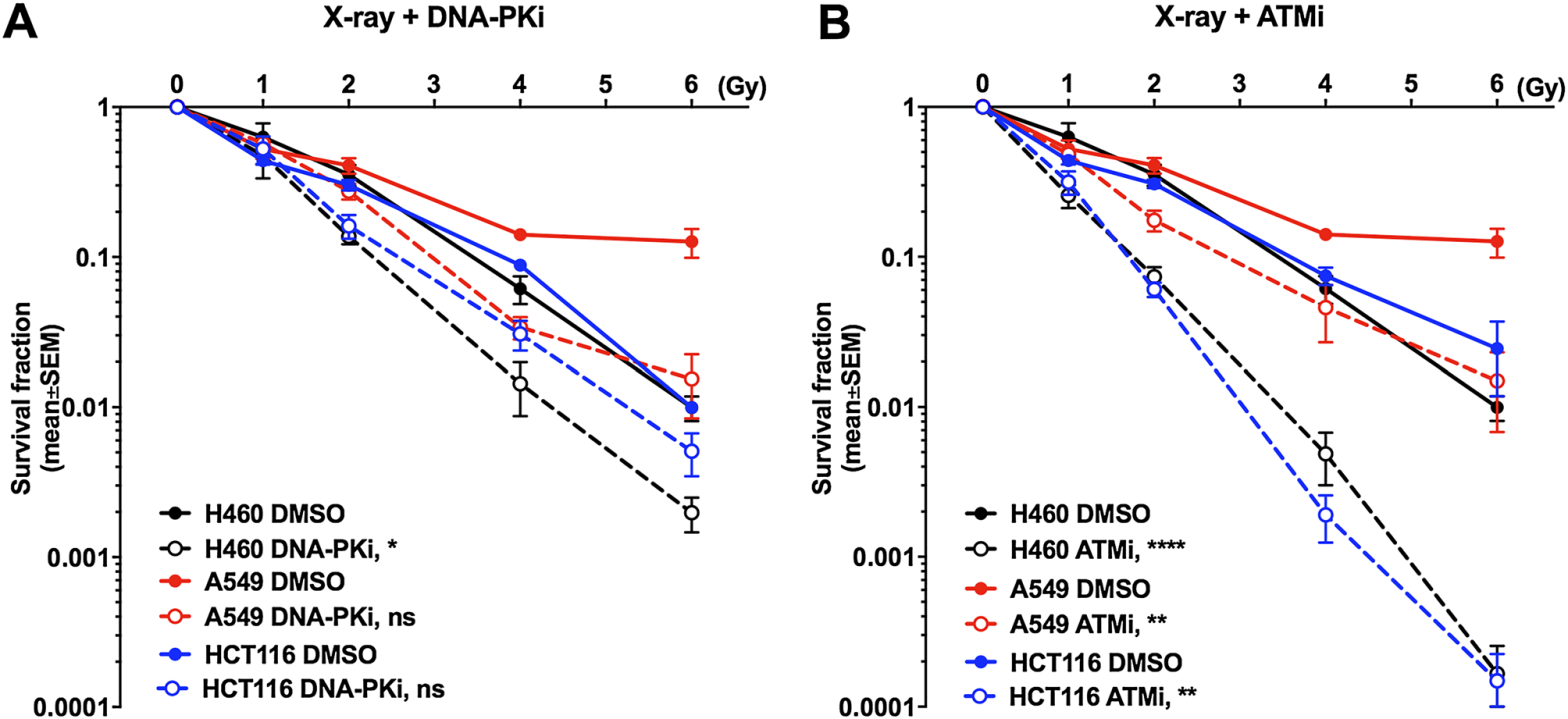
DDR inhibitors sensitized cancer cells to α-radiation. Clonogenic survival for DNA-PKi (**A**) or ATMi (**B**). H460, A459, HCT116 and HEK293 cells were treated with 200 nM DNA-PKi and ATMi for 2 h prior to α-radiation, giving doses of 4, 8, 16 Gy, then incubated for 24 h and reseeded for colony formation. Statistical analysis: mean ± SEM, n ≥ 2, 2way ANOVA, Tukey's multiple comparisons test, (*) p < 0.05, (**) p < 0.01.

**Figure 3:**
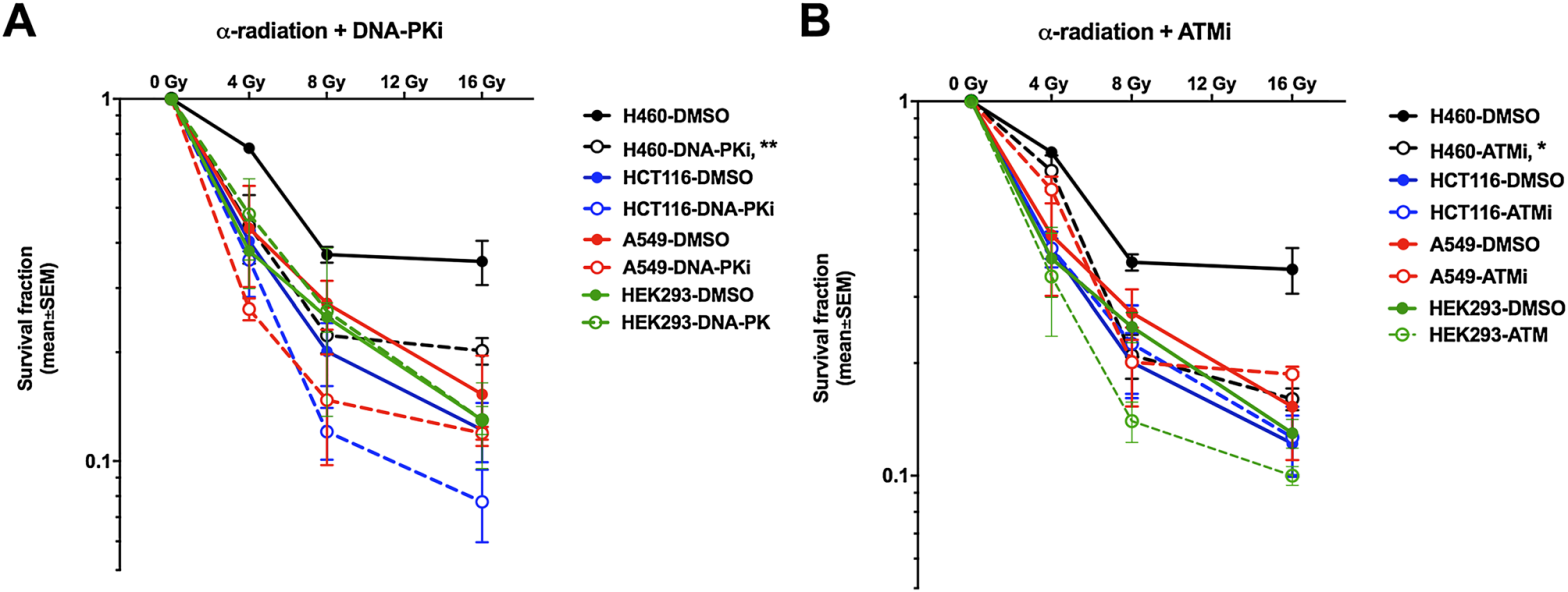
DDR inhibitors sensitized cancer cells to X-ray radiation. Clonogenic survival after combining DNA-PKi (200 nM) and radiation (**A**), ATMi (200 nM) and radiation (**B**). The cells were treated 2 h prior to radiation, then incubated for 24 h and reseeded for colony formation. Statistical analysis: 2way ANOVA, Tukey's multiple comparisons test; (*) p < 0.05, (**) p < 0.01, (****) p < 0.0001.

Aiming to elucidate the functions of two main kinases in DDR induced by alpha radiation and X-ray as control, cell survival was determined in several cancer cell lines. H460, A549, HCT116 and HEK293 were treated with the same concentration of DNA-PKi and ATMi (200 nM) in combination with α-radiation. The transwell system was used to deliver doses of 4, 8 and 16 Gy (Fig. 2). In H460 cells α-radiation in combination with DNA-PKi or ATMi led to a statistically significant reduction of the survival fraction. The other cell lines did show a tendency to increased radiosensitivity, which, however, did not reach significance. The combination of DNA-PKi with α-radiation only slightly reduced the survival in HCT116 and A549 cancer cell lines from 0.12 to 0.08% in HCT116 and from 0.15 to 0.12% in A549 at 16 Gy (Fig. 3) The combination of X-ray radiation with ATMi or DNA-PKi showed good radiosensitizing effect in all cancer cell lines. Whereas DNA-PKi had sensitizing effects together with alpha radiation on the cancer cell, panel immortialized HEK293 cells did not show any additive effect for DNA-PKi. In contrast, ATMi increased the sensitivity of HEK293 cells towards alpha radiation. Interestingly, there seems to be a trend that the addition of the DDR inhibitors increased the effect of alpha radiation at lower doses while with increasing dose the sensitizing effect leveled out.

Overall, the combination of DNA-PKi with α-radiation most efficiently reduced the survival of H460, A549 and HCT116 cells. In contrast, HEK293 cells lines were more sensitive to combination of ATMi and α-radiation.

Additionally, we calculated the sensitization enhancement ratio (SER) for alpha radiation at 8 Gy and X-ray at 4 Gy (Table 2). In general, the SER (8 Gy, α) values were smaller than SER (4 Gy, X-ray) values after DNA-PKi and ATMi in all cell lines. Interestingly, SER (8 Gy, α) value for HEK293 was higher after ATMi treatment than DNA-PKi (0.96; 1.79), in contrast to HCT116 (1.67; 0.89). The SER (8 Gy, α) values were similar for H460 and A549 cells. In contrast, SER (8 Gy, X-ray) was around 10 times higher after ATMi (39.2) as compared to DNA-PKi (2.87) in HCT116. Overall ATMi treatment showed stronger sensitization effect with X-ray as compared to DNA-PKi. However, the results showed that combining of ATM and DNA-PK inhibitors with α-radiation in cancer cell as well as normal HEK293 cell line sensitized to a lower degree as compared to combination with X-rays.

**Table 2:** The sensitization enhancement ratio after alpha radiation and X-ray exposure.

	H460	A549	HCT116
**SER**_**alpha**_** at 8 Gy**			
DNA-PKi	1.6	1.85	1.67
ATMi	1.78	1.36	0.89
**SER**_**X-ray**_** at 4 Gy**			
DNA-PKi	4.29	4.15	2.87
ATMi	12.62	3.08	39.2

### Increased number of micronuclei after combined treatment with DDR inhibitors and irradiation

**Figure 4:**
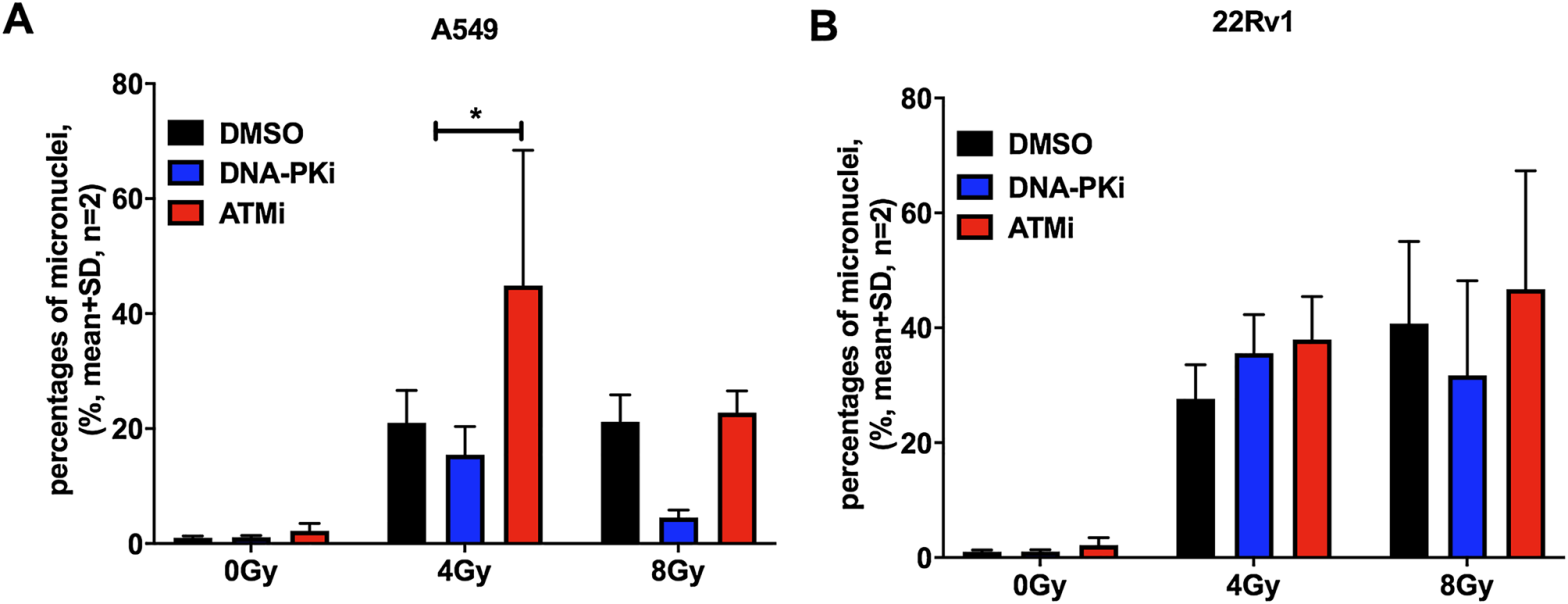
ATM inhibitor increased the formation of micronuclei after X-ray radiation in A549 (**A**) and in 22Rv1 (**B**). A549 and 22Rv1 cancer cell lines were treated with 200 nM DNA-PKi or ATMi for 2 h prior to X-ray exposure of 4 and 8 Gy, then were incubated an additional 24 h (48 h for 22Rv1) before analysis; statistical analysis: 2way ANOVA, Dunnett's multiple comparisons test: mean + SD, n = 2, (*) p < 0.05.

A variety of genotoxic agents like radiation, chemotherapeutics and DDR inhibitors may induce the formation of micronuclei potentially leading to cell death. In agreement, many cytotoxic chemotherapeutics interfering with DNA replication and/or transcription such as cyclophosphamide, cytarabine, mitomycin C are recommended as positive controls in the micronucleus test (OECD TG 487, 2016). Therefore, the genotoxic effect of a combination of different types of radiation and DNA repair inhibitors on the formation of micronuclei in cancer and immortalized cell lines was investigated. H460, H1299, A549, 22Rv1 and HEK293 cells were treated with DNA-PKi (200 nM) or ATMi (200 nM) 2 h prior to X-ray exposure of 4 or 8 Gy or to α-radiation of 4, 8 and 16 Gy (1.3 kBq/cm^2^ for 2, 4 and 8 h). As shown in Fig. 4, the micronucleus frequency was increased in H460 and 22Rv1 cancer cell lines after exposure to X-ray at doses of 4 and 8 Gy alone. The combination of X-ray irradiation and the ATM inhibitor significantly increased the formation of micronuclei at 4 Gy in A459 cells as compared to radiation alone. Interestingly, we observed a significantly less pronounced formation of micronuclei in combination of radiation and DNA-PKi at 4 and 8 Gy as compared to radiation alone in A549 as well as in 22Rv1 at 8 Gy.

**Figure 5:**
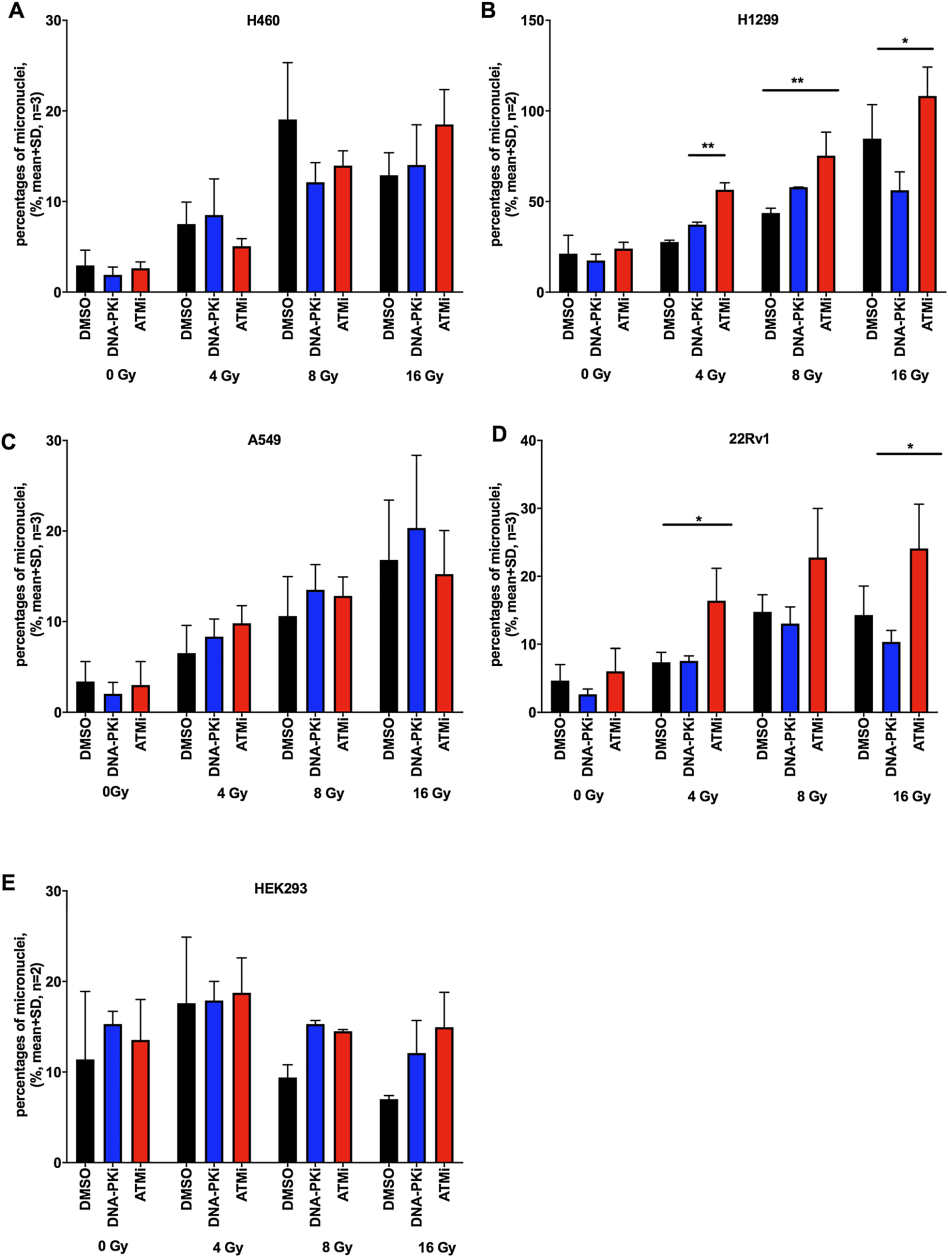
DDR inhibitors increased the formation of micronuclei after α-radiation in several cell lines. The micronucleus frequency for H460 cells (**A**), 22Rv1 (**B**), H1299 (**C**) and A549 (**D**) and HEK293 (**E**) treated with 200 nM DNA-PKi or ATMi for 2 h prior to α-radiation with activity of 1.3 kBq/cm^2^ for 2, 4, 8 h, yielding doses of 4, 8 and 16 Gy incubated an additional 24 h (48 h for 22Rv1) before analysis; statistical analysis: 2way ANOVA, Dunnett's multiple comparisons test: mean + SD, n ≥ 2, (*) p < 0.05, (**) p < 0.01.

Similar studies were performed in order to investigate the formation of micronuclei in response to the combination of DDR inhibitors and α-radiation. ATMi treatment with α-radiation together significantly induced the formation of micronuclei in 22Rv1 cells as well as in H1299 cells (Fig. 5). Unexpectedly, the combination of α-radiation with the DNA-PKi did not elicit the formation of micronuclei in the cancer cell lines. In contrast, HEK293 cells showed an increased micronucleus frequency after DNA-PKi and after ATMi treatment in combination with α-radiation at 8 and 16 Gy as compared to radiation alone. Furthermore, unirradiated cells showed a high number of spontaneously formed micronuclei. Moreover, we did not observe a dose–response effect in the DMSO group in HEK293.

Overall, the combination of α-radiation and ATM inhibitor had a significantly stronger genotoxic effect as compared to α-radiation alone but was similar to results obtained with X-ray radiation.

### Effects of combining α-radiation with DNA-PKi and ATMi on cell cycle distribution in cancer and normal cells

**Figure 6:**
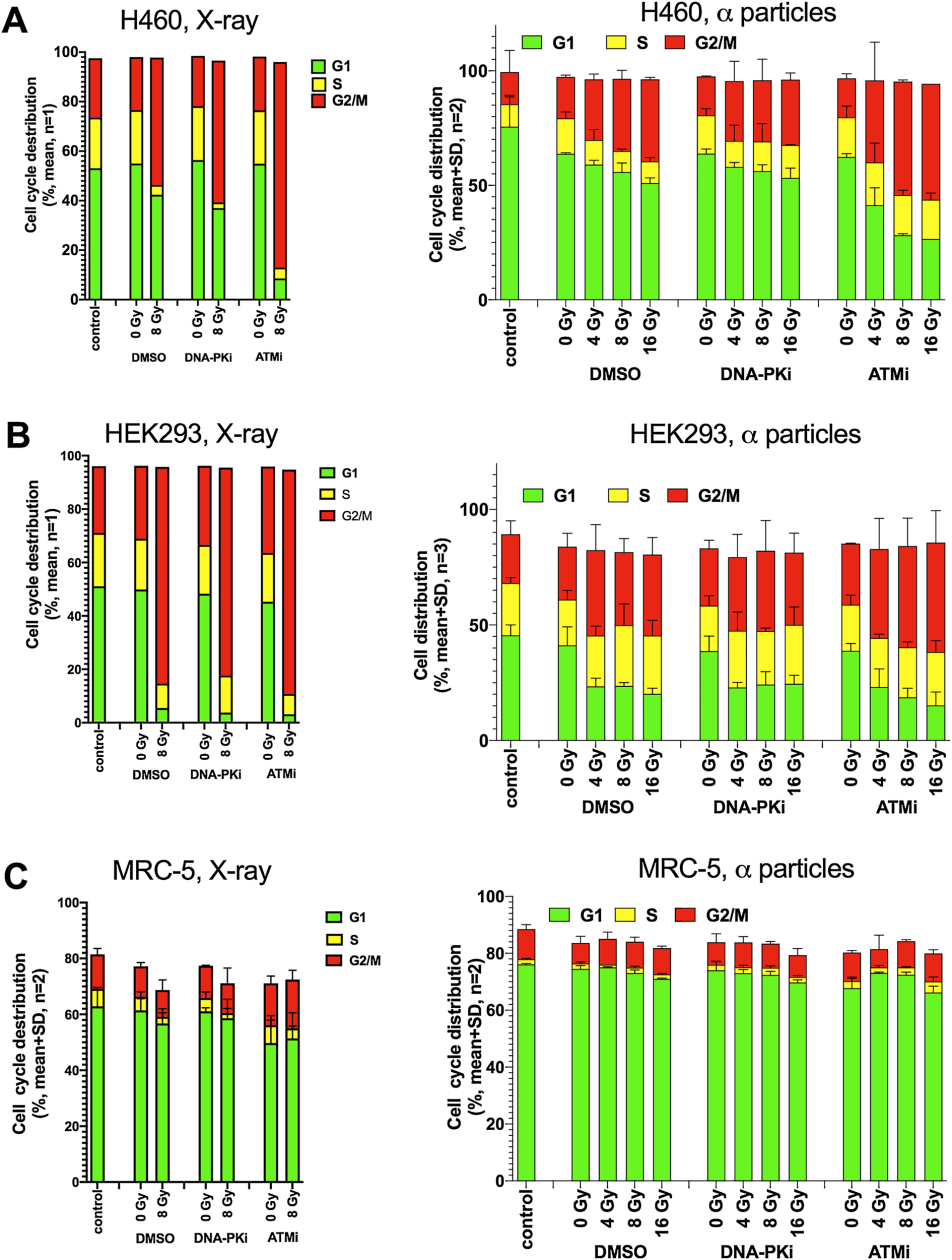
DDR inhibitors selectively affect cell cycle arrest in G_2_/M after X-ray and alpha radiation in various cell lines. Cell cycle distributions for H460 (**A**), HEK293 (**B**) and MRC-5 (**C**) cells after treatment with 200 nM DNA-PKi M3814 and ATMi ADZ-1390 for 2 h prior to X-ray exposure or alpha radiation.

To investigate the effects of α-and X-ray radiation after DDR inhibitor treatment on cell cycle distribution cells (H460, HEK293, MRC-5, IMR-90 and CCD 1096Sk) were treated with DNA-PKi or ATMi (200 nM) 2 h before irradiation. The cells were α-irradiated for 2 h with different doses of 4, 8 and 16 Gy, or with X-ray at 8 Gy. Results after X-ray radiation showed that DNA-PKi and ATMi treatments strongly reduced the number of cells in S phase and caused an increasing number of cells in G2/M phase. This effect on the X-ray-radiation-induced cell cycle arrest in G2/M phase was similar in H460 cancer cells and immortalized HEK293 cells (Fig. 6A,B). However, DNA-PKi and ATMi treatments did not induce a G_2_/M arrest in primary MRC-90 fibroblasts (Fig. 6C), IMR-90 and CCD 1096Sk (see “Suppl.”).

Upon α-radiation H460 and HEK293 cells showed an increasing number of cells in G2/M phase but no reduction in the number of cells in S phase. ATMi treatment in combination with α-radiation more strongly arrested H460 and HEK293 cells in G_2_/M phase as compared DNA-PKi treatment and α-radiation alone. MRC-5 fibroblasts did not show any effect of ATMi and DNA-PKi treatments with α-radiation in 24 h.

Overall, both radiation treatments strongly activated the G_2_/M checkpoint in H460 and HEK293 cells, additional treatment with ATMi increased this effect. The primary fibroblasts did not show an arrest in G_2_/M phase after both types of radiation in combination with DDR inhibitors, although a reduction of proliferation after α-radiation in primary fibroblasts was clearly visible (see “Suppl.”). It seemed that α-radiation may less affect the G_2_/M checkpoint but rather activate other checkpoints in primary fibroblasts.

## Discussion

High LET α-particles deposit a much greater fraction of total energy in a shorter distance in the cells as low LET X-ray radiation. This is manifested by a high relative biological effectiveness (RBE). Therefore, fewer nuclear hits are required to kill a cell via unrepaired complex DNA damage. However, normal tissue effects after radiation are caused by a combination of cell death and other processes such as cell differentiation, epithelial to mesenchymal transition, proliferation of specific cells, extracellular matrix deposition, and many other reactions^21,22^. Although, high LET radiation generates more complicated and varied cellular effects alone, in the present study we investigated the combined effect of α- or X-ray radiation with DDR inhibitors on cell survival and formation of micronuclei in cancer and normal cells.

Previous studies have widely shown the combined effect of different DDR inhibitors with low LET radiation on cell survival in tumor cells^23,24^. In our study we choose DNA-PKi (M3814)^25^ and ATMi (AZD-1390), which are currently investigated in clinical studies in monotherapy as well as in combination treatments, and investigated different endpoints after the combination with α-radiation or X-ray in vitro*.* DNA-PK is one of the central constituents of DDR, and its inhibition was shown to radiosensitize NSCLC cell lines (NU7441 at 0.3 µM)^26^, or human osteosarcoma cells (KU60648, 0.3 µM)^27^ to X-ray radiation as well as to carbon ion exposure (M3814 at 0.1–1 µM)^24^. Our results are in line with these studies as the strong sensitizing effect of M3814 (at 0.2 µM) to X-ray radiation decreased cell survival of almost all observed cancer cells (except A549). Moreover, Sunada et al.^26^, reported that NU7441 induced higher radiosensitization with X‐rays (1.94) than carbon ions (1.58) in H1299 cells, and the degree of sensitization was smaller 1.77, 1.55 for A549 cells. Althought, carbon ions showed the relative biological effectiveness (RBE) of 2.15 in A549 cells and 1.87 in H1299 cells. Here we showed that DNA-PK inhibitor was less effective in combination with α-radiation. The reduced level of DNA-PK autophosphorylation by M3814 suggests that DNA-PK-dependent DSB repair by NHEJ is inhibited in cancer cells. Also the HR repair pathway utilized more in DSB induced by high LET radiation^28,29^. Takahashi et al.^30^ reported that when using HR and NHEJ pathway‐deficient mouse embryonic fibroblasts (*p53*^−/−^ MEF *Lig4*^+/+^ *Rad54*^−/−^; *Lig4*^−/−^ *Rad54*^+/+^; *Lig4*^−/−^ *Rad54*^−/−^), high LET radiation caused significant radiosensitization even in NHEJ‐defective cells. Our results showed here that α-radiation induced unrepaired complexes DNA damage in cancer cells as well in HEK293 and additionally reduction of NHEJ pathway has a small effect on cell survival. The ATM inhibitor also strongly sensitized all cancer cell lines investigated here to X-ray radiation; however, only H460 was significantly sensitized to α-radiation. The SER was higher for ATMi (1.79) and DNA-PKi (0.96) in HEK293 cells. Moreover, a recent clinical study showed that there is a subgroup of patients with resistence to prostate-specific membrane antigen targeted alpha therapy (PSMA-TAT)^31^. The patients had seven deletrerious mutation of *ATM*, *CHEK*2, or *TP53*. Taking together that DNA-PK and ATM inhibitors showed less radiosensitizing effect on cell survival after α-radiation than after X-ray exposure in cells. Therefore combination therapies of TAT and ATM inhibitors might act complementarily.

Several studies have shown that α-radiation induced a higher micronucleus frequency than X-ray exposure in human peripheral blood lymphocytes^32,33^. In this study we observed significantly increased micronucleus frequency after co-treatment of α-radiation with ATM inhibitors in H1299 and 22Rv1 cancer cell lines. Both inhibitors (DNA-PK and ATM) in combination with alpha radiation had the similar effect on the formation of micronuclei in HEK293. Although the cancer cell lines display a priori a higher MN background, the effect of co-treatment with α-radiation and DNA-repair inhibitors is much stronger than α-radiation alone. It suggests that the DNA damage after co-treatment is much more complex in the nuclei as a result of unrepaired or mis-repaired DNA breaks, and it potentially leads to the reduction of cell survival, cell death, or cell senescence.

Additionally, our results showed that ATM inhibitor had a stronger effect on the cell cycle arrest in G_2_/M phase for both radiation qualities. The HEK293 cells showed the greater sensitivity than cancer cells. Interestingly, immortalized HEK293 cells had the similar endpoints effects as cancer cells. One of the remarkable effects was that primary fibroblasts did not show any response on cell cycle arrest in combination with DNA-PKi and ATMi, at least at 24 h after irradiation. Perhaps, the cells are in G_0_ phase, which is less radiosensitive or alpha irradiated fibroblasts showed late-effects like higher number of apoptotic at 144 h as compared after X-ray radiation^34^. However, they reduced the number of cells at 5 days after alpha irradiation (see “Suppl.”). The high-LET radiation may not only differ from low-LET radiation in complexity DNA damage induction and cell death, but also in the type of mechanisms it activates. Moreover, different organs respond to radiation by activating specific mechanisms, which results in tissue-specific manifestations of normal tissue toxicity like pneumonitis and fibrosis^35,36^.

## Conclusion

In this work we compare the potential advantage of the combination DNA repair inhibitors with α-radiation or X-ray radiation to sensitize cells The most promising inhibitor in combination with alpha radiation was the ATM inhibitor and its reduction of cell survival by increasing the formation of micronuclei in cancer cells. In future studies we will extend this concept to investigate the combined effect of DNA-PK and ATM inhibitor and α-radiation in vivo.

## Supplementary Information

Supplementary Information.
