# Supplementary material for: DNA repair inhibitors sensitize cells differently to high and low LET radiation
Source: Sci Rep. 2021 Dec 1;11:23257. doi: 10.1038/s41598-021-02719-9 (PMC8636489; doi:10.1038/s41598-021-02719-9)
Supplement: Supplementary file 1 — Supplementary Information. [file 41598_2021_2719_MOESM1_ESM.docx]

# DNA repair inhibitors sensitize cells differently to high and low LET radiation

Kristina Bannik^1^, Balázs Madas ^2^, Sabrina Jarke^1,3^, Andreas Sutter^1^, Gerhard Siemeister^1,3^, Christoph Schatz^1^, Dominik Mumberg^1^, Sabine Zitzmann-Kolbe^1^.

^1^Bayer AG, Pharmaceuticals Division, Berlin, Germany

^2^ Centre for Energy Research, Budapest, Hungary

^3^ Present address: Nuvisan-ICB GmbH, Berlin, Germany

# Supplementary material

**Supplementary material figure 1**


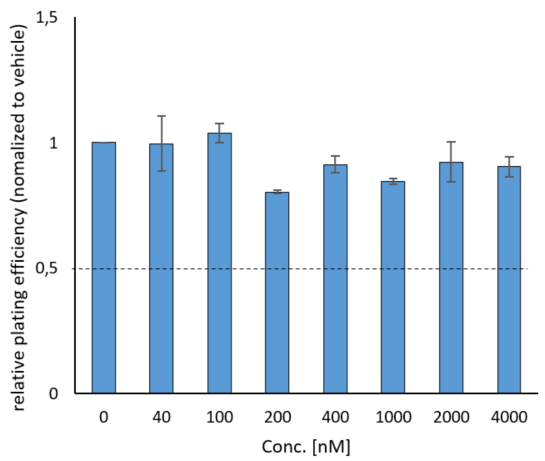


**Cellular toxicity of different DDRis in H460 lung cancer cells.** Cellular toxicity of the ATM inhibitor (ATMi) AZD1390 (left) and the DNA-PK inhibitor (DNA-PKi) M3814 (right) in H460 cells was determined using the clonogenic survival assay. Colonies containing more than 50 cells were scored. Data represent mean values with standard error from two experiments (ATMi) and from a single experiment (DNA-PKi).

**Supplementary material figure 2**

**Cell cycle distribution after X-ray or alpha irradiation in IMR-90 and CDD 1096SK cells.**

**
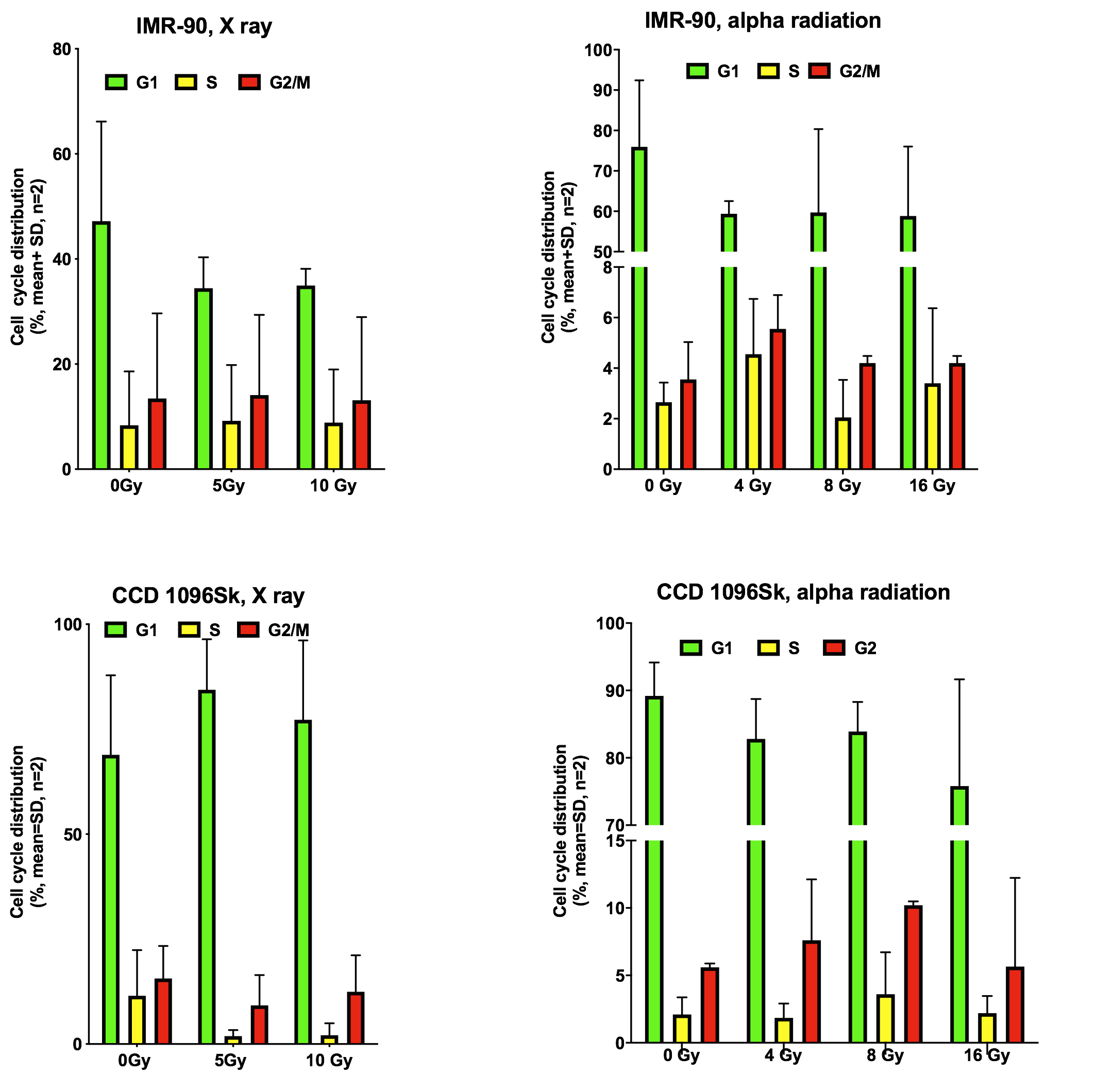
**

**Supplementary material figure 3**

**Proliferation Assays**

The cells (H460, HEK293, MRC-5 and CCD 1096Sk) were plated into Transwell inserts (#3460, Corning) with a density of 150.000 cells/well, next day irradiated with alpha radiation, activity of 5, 10, 20 kBq for 2, 4, 8 hrs. The cells were incubated 24 h and were plated out at a density of 4000 (H460, HEK293) or 8000 (MRC-5, CCD 1096Sk) cells/measurement point in a 96-well microtiter plate in 200 μL of growth medium. After continuous incubation for 5 d at 37 °C, the cells were fixed with glutaraldehyde, stained with crystal violet (Kueng, W.; Silber, E.; Eppenberger, U. Quantification of cells cultured on 96-well plates. Anal. Biochem. 1989, 182, 16−19) and the absorbance was recorded at 595 nm using Tecan Sunrise equipment (Tecan Group Ltd., Switzerland). All measurements were performed in centuplicate.


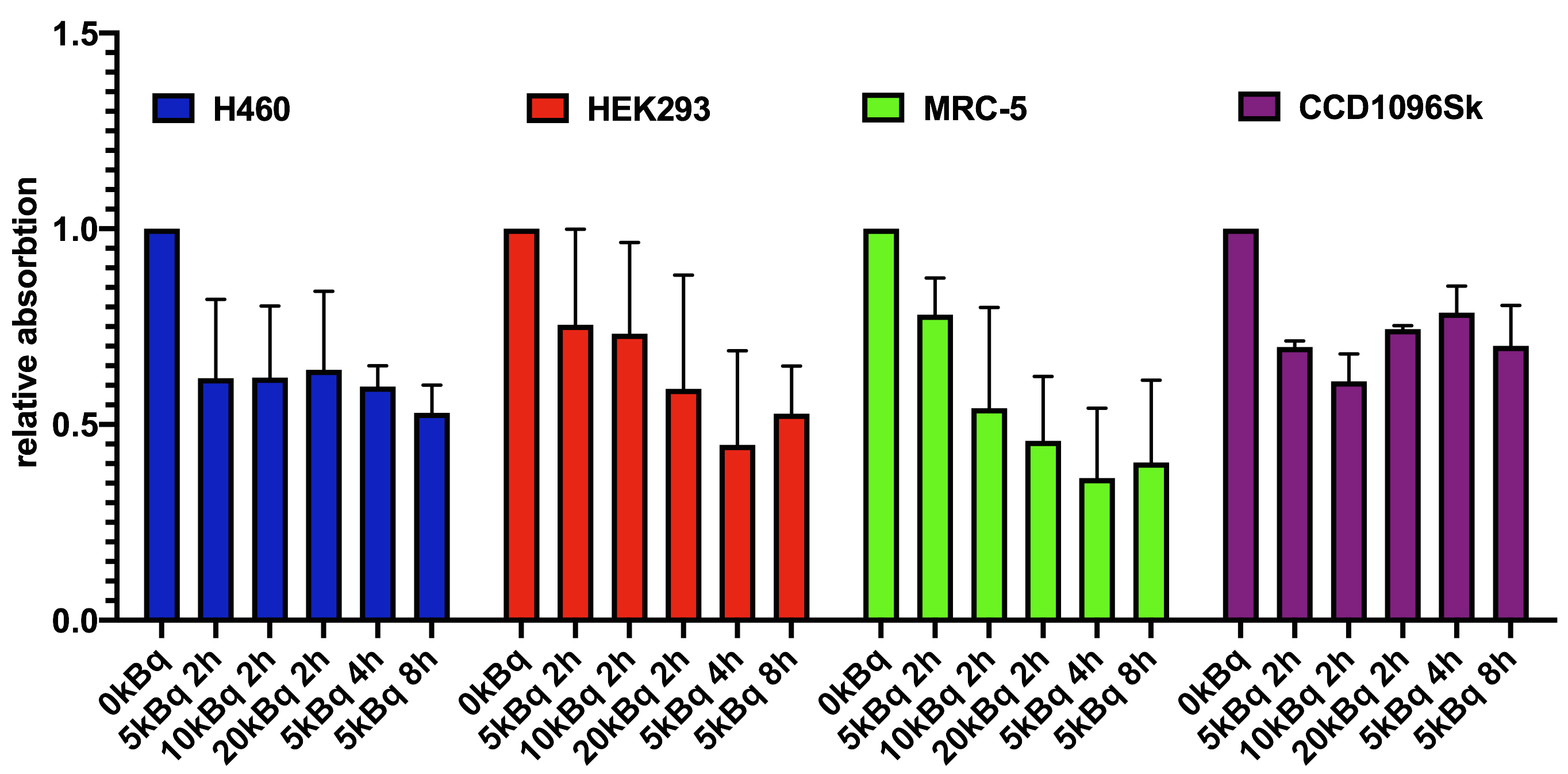


**Supplementary material figure 4**

**A**


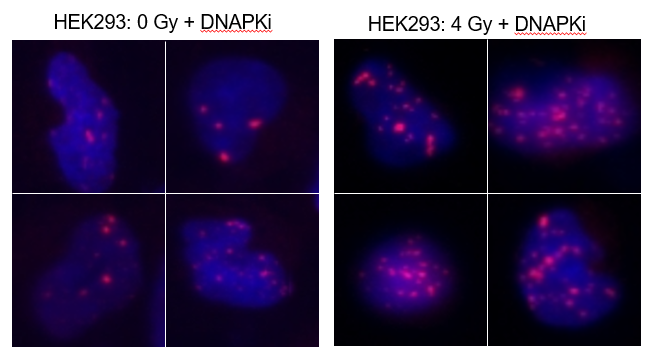


The cells (HEK293) were plated into Transwell inserts and subject to DNAPKi treatment at 200 nM. Next day the cells were irradiated with alpha radiation until a dose of 4 Gy. The cells were incubated 24 h and then stained with DAPI (blue) and 53BP1 (red) antibody for DNA-damage.

**B**


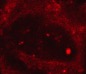

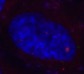


**overlap**

**gH2AX**

**53PB1**

**DAPI**

**HEK293 0 Gy**


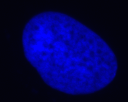

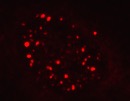

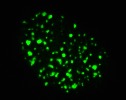

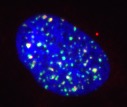


**HEK293 10 Gy X-ray**


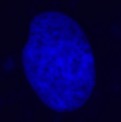

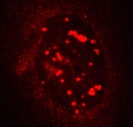

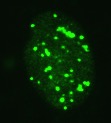

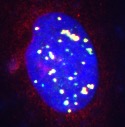


**HEK293 8 Gy Ra-223**

The cells (HEK293) were plated into Transwell inserts. Next day the cells were irradiated with X-ray or Ra-223 until a dose of 0, 8 or 10 Gy. The cells were incubated 24 h and then stained with 53BP1 (red), gH2AX (green), DAPI (blue) antibody for DNA-damage.
